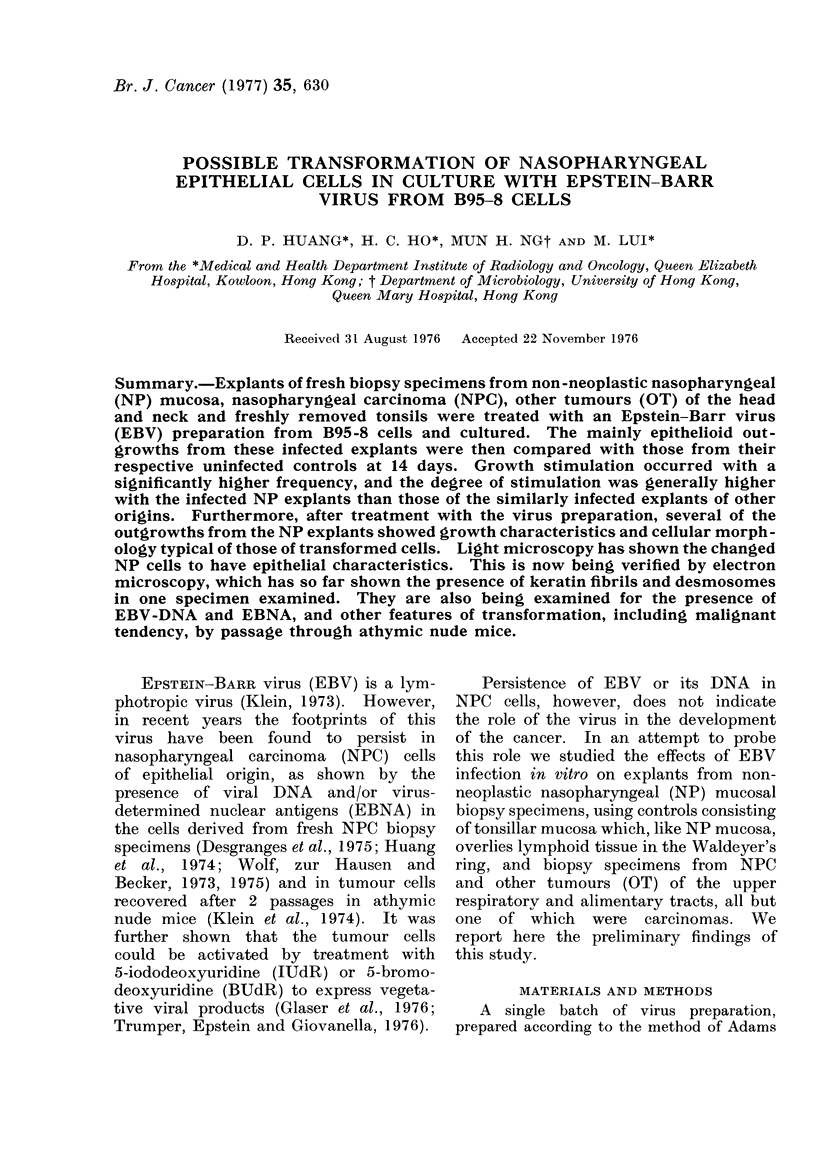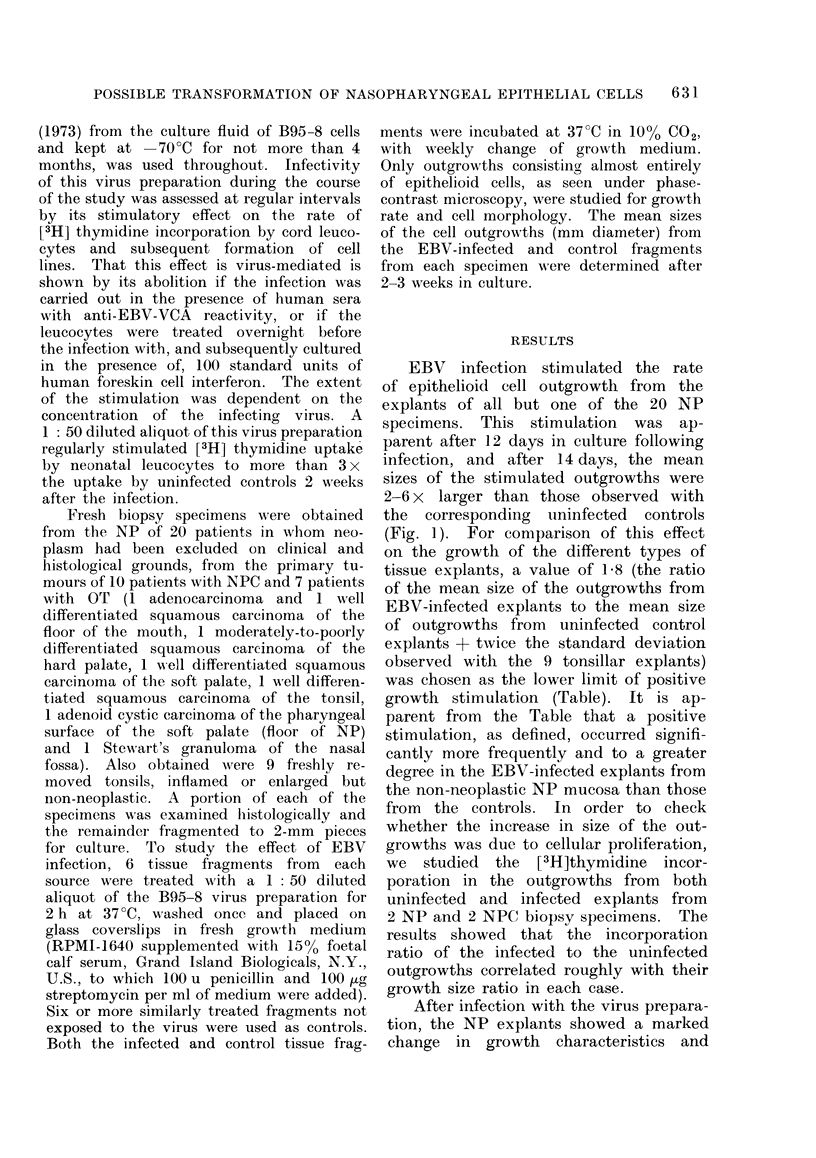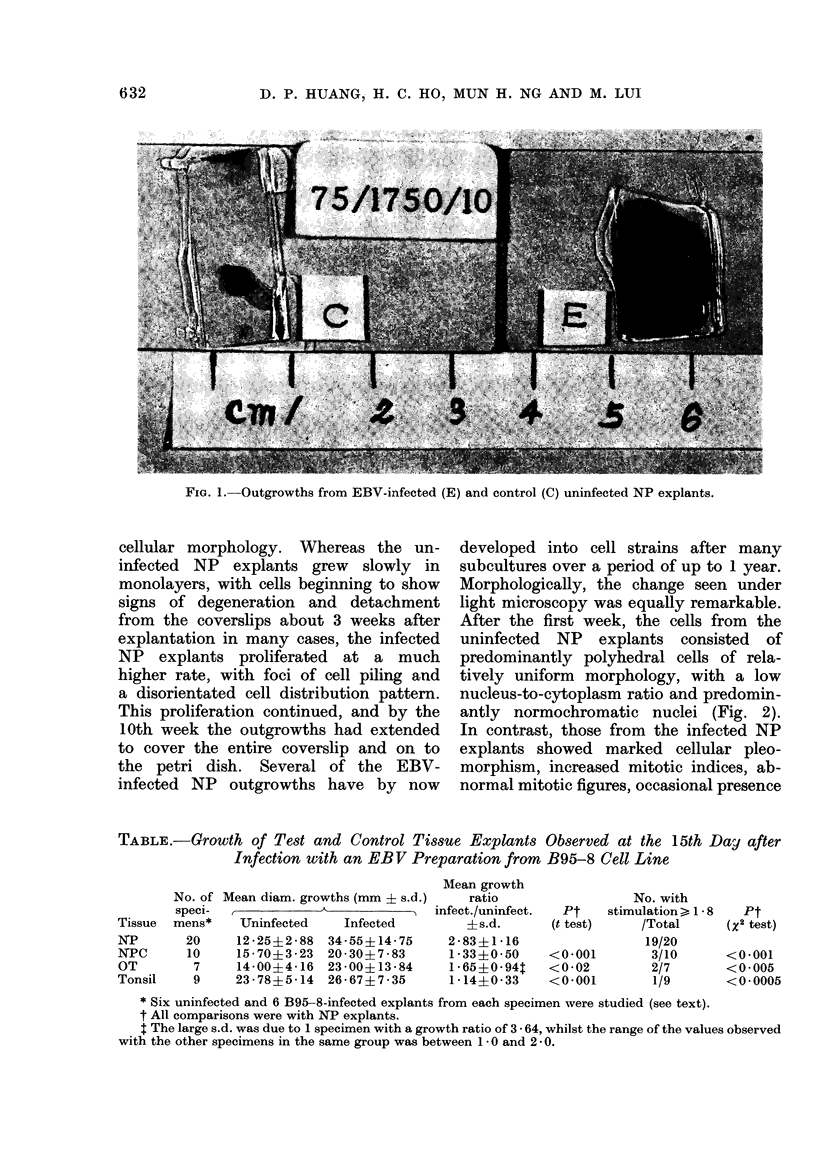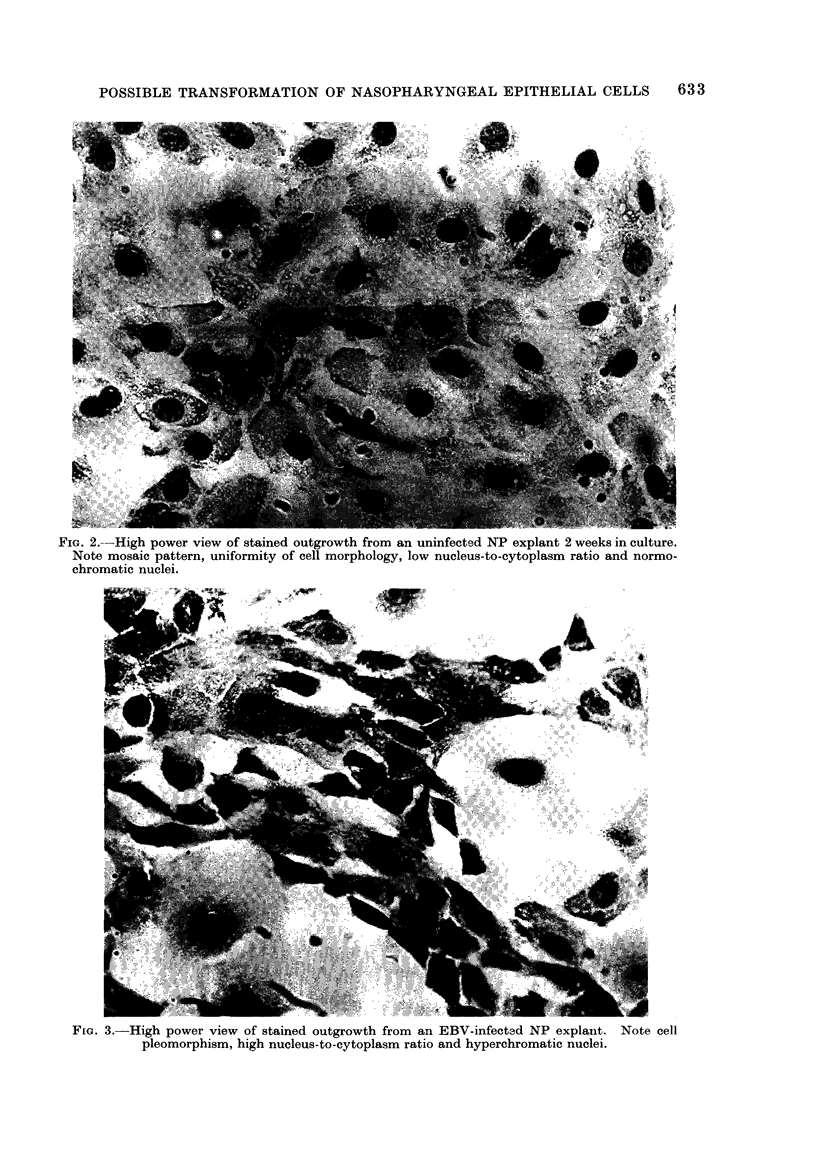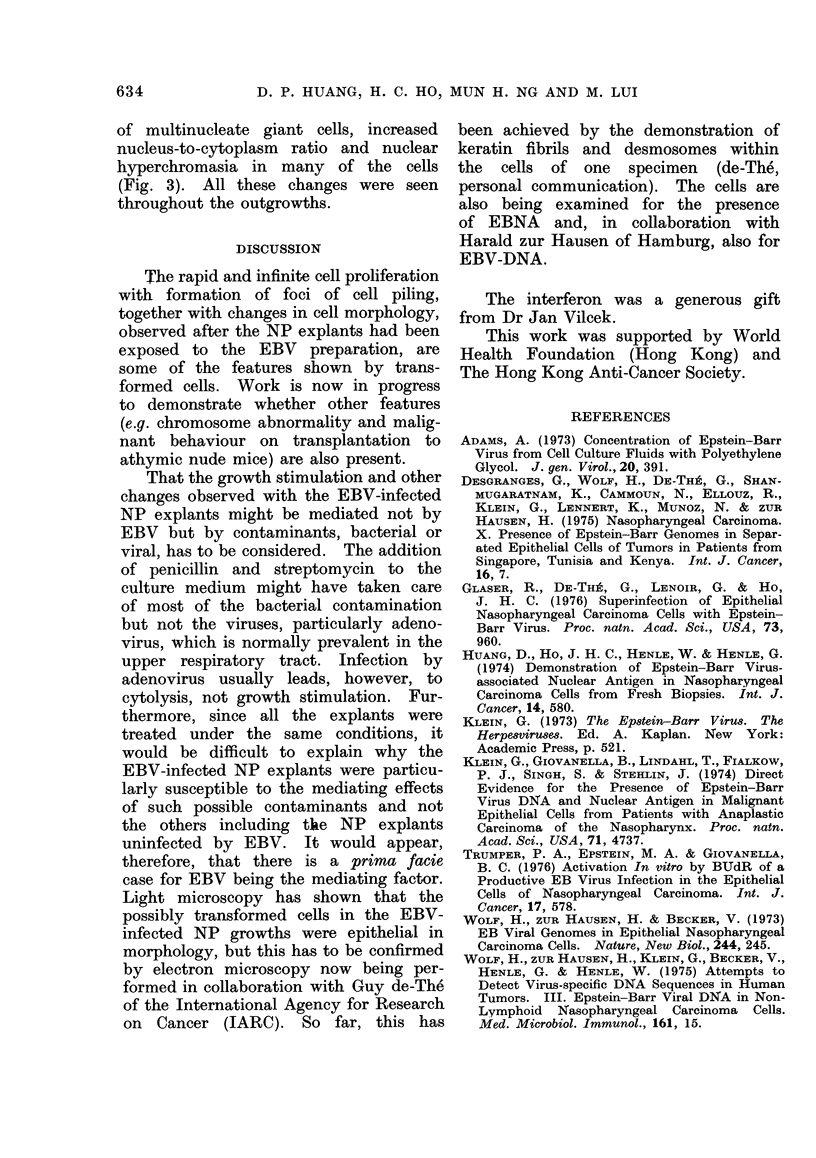# Possible transformation of nasopharyngeal epithelial cells in culture with Epstein-Barr virus from B95-8 cells.

**DOI:** 10.1038/bjc.1977.98

**Published:** 1977-05

**Authors:** D. P. Huang, H. C. Ho, M. H. Ng, M. Lui

## Abstract

**Images:**


*Br. J. Cancer* (1977) **35**, 630

### POSSIBLE TRANSFORMATION OF NASOPHARYNGEAL EPITHELIAL CELLS IN CULTURE WITH EPSTEIN–BARR VIRUS FROM B95–8 CELLS

D. P. HUANG*, H. C. HO*, MUN H. NG† AND M. LUI*

*From the *Medical and Health Department Institute of Radiology and Oncology, Queen Elizabeth Hospital, Kowloon, Hong Kong; † Department of Microbiology, University of Hong Kong, Queen Mary Hospital, Hong Kong*

Received 31 August 1976 Accepted 22 November 1976

**Summary.—Explants of fresh biopsy specimens from non-neoplastic nasopharyngeal (NP) mucosa, nasopharyngeal carcinoma (NPC), other tumours (OT) of the head and neck and freshly removed tonsils were treated with an Epstein–Barr virus (EBV) preparation from B95-8 cells and cultured. The mainly epithelioid outgrowths from these infected explants were then compared with those from their respective uninfected controls at 14 days. Growth stimulation occurred with a significantly higher frequency, and the degree of stimulation was generally higher with the infected NP explants than those of the similarly infected explants of other origins. Furthermore, after treatment with the virus preparation, several of the outgrowths from the NP explants showed growth characteristics and cellular morphology typical of those of transformed cells. Light microscopy has shown the changed NP cells to have epithelial characteristics. This is now being verified by electron microscopy, which has so far shown the presence of keratin fibrils and desmosomes in one specimen examined. They are also being examined for the presence of EBV-DNA and EBNA, and other features of transformation, including malignant tendency, by passage through athymic nude mice.**

Epstein–Barr virus (EBV) is a lymphotropic virus (Klein, 1973). However, in recent years the footprints of this virus have been found to persist in nasopharyngeal carcinoma (NPC) cells of epithelial origin, as shown by the presence of viral DNA and/or virus-determined nuclear antigens (EBNA) in the cells derived from fresh NPC biopsy specimens (Desgranges *et al.*, 1975; Huang *et al.*, 1974; Wolf, zur Hausen and Becker, 1973, 1975) and in tumour cells recovered after 2 passages in athymic nude mice (Klein *et al.*, 1974). It was further shown that the tumour cells could be activated by treatment with 5-iododeoxyuridine (IUdR) or 5-bromodeoxyuridine (BUdR) to express vegetative viral products (Glaser *et al.*, 1976; Trumper, Epstein and Giovanella, 1976).

Persistence of EBV or its DNA in NPC cells, however, does not indicate the role of the virus in the development of the cancer. In an attempt to probe this role we studied the effects of EBV infection *in vitro* on explants from non-neoplastic nasopharyngeal (NP) mucosal biopsy specimens, using controls consisting of tonsillar mucosa which, like NP mucosa, overlies lymphoid tissue in the Waldeyer's ring, and biopsy specimens from NPC and other tumours (OT) of the upper respiratory and alimentary tracts, all but one of which were carcinomas. We report here the preliminary findings of this study.

### MATERIALS AND METHODS

A single batch of virus preparation, prepared according to the method of Adams

(1973) from the culture fluid of B95-8 cells and kept at −70°C for not more than 4 months, was used throughout. Infectivity of this virus preparation during the course of the study was assessed at regular intervals by its stimulatory effect on the rate of [$^3$H] thymidine incorporation by cord leucocytes and subsequent formation of cell lines. That this effect is virus-mediated is shown by its abolition if the infection was carried out in the presence of human sera with anti-EBV-VCA reactivity, or if the leucocytes were treated overnight before the infection with, and subsequently cultured in the presence of, 100 standard units of human foreskin cell interferon. The extent of the stimulation was dependent on the concentration of the infecting virus. A 1 : 50 diluted aliquot of this virus preparation regularly stimulated [$^3$H] thymidine uptake by neonatal leucocytes to more than 3× the uptake by uninfected controls 2 weeks after the infection.

Fresh biopsy specimens were obtained from the NP of 20 patients in whom neoplasm had been excluded on clinical and histological grounds, from the primary tumours of 10 patients with NPC and 7 patients with OT (1 adenocarcinoma and 1 well differentiated squamous carcinoma of the floor of the mouth, 1 moderately-to-poorly differentiated squamous carcinoma of the hard palate, 1 well differentiated squamous carcinoma of the soft palate, 1 well differentiated squamous carcinoma of the tonsil, 1 adenoid cystic carcinoma of the pharyngeal surface of the soft palate (floor of NP) and 1 Stewart's granuloma of the nasal fossa). Also obtained were 9 freshly removed tonsils, inflamed or enlarged but non-neoplastic. A portion of each of the specimens was examined histologically and the remainder fragmented to 2-mm pieces for culture. To study the effect of EBV infection, 6 tissue fragments from each source were treated with a 1 : 50 diluted aliquot of the B95-8 virus preparation for 2 h at 37°C, washed once and placed on glass coverslips in fresh growth medium (RPMI-1640 supplemented with 15% foetal calf serum, Grand Island Biologicals, N.Y., U.S., to which 100 u penicillin and 100 $\mu$g streptomycin per ml of medium were added). Six or more similarly treated fragments not exposed to the virus were used as controls. Both the infected and control tissue fragments were incubated at 37°C in 10% $CO_2$, with weekly change of growth medium. Only outgrowths consisting almost entirely of epithelioid cells, as seen under phase-contrast microscopy, were studied for growth rate and cell morphology. The mean sizes of the cell outgrowths (mm diameter) from the EBV-infected and control fragments from each specimen were determined after 2–3 weeks in culture.

### RESULTS

EBV infection stimulated the rate of epithelioid cell outgrowth from the explants of all but one of the 20 NP specimens. This stimulation was apparent after 12 days in culture following infection, and after 14 days, the mean sizes of the stimulated outgrowths were 2–6× larger than those observed with the corresponding uninfected controls (Fig. 1). For comparison of this effect on the growth of the different types of tissue explants, a value of 1·8 (the ratio of the mean size of the outgrowths from EBV-infected explants to the mean size of outgrowths from uninfected control explants + twice the standard deviation observed with the 9 tonsillar explants) was chosen as the lower limit of positive growth stimulation (Table). It is apparent from the Table that a positive stimulation, as defined, occurred significantly more frequently and to a greater degree in the EBV-infected explants from the non-neoplastic NP mucosa than those from the controls. In order to check whether the increase in size of the outgrowths was due to cellular proliferation, we studied the [$^3$H]thymidine incorporation in the outgrowths from both uninfected and infected explants from 2 NP and 2 NPC biopsy specimens. The results showed that the incorporation ratio of the infected to the uninfected outgrowths correlated roughly with their growth size ratio in each case.

After infection with the virus preparation, the NP explants showed a marked change in growth characteristics and

FIG. 1.—Outgrowths from EBV-infected (E) and control (C) uninfected NP explants.

cellular morphology. Whereas the uninfected NP explants grew slowly in monolayers, with cells beginning to show signs of degeneration and detachment from the coverslips about 3 weeks after explantation in many cases, the infected NP explants proliferated at a much higher rate, with foci of cell piling and a disorientated cell distribution pattern. This proliferation continued, and by the 10th week the outgrowths had extended to cover the entire coverslip and on to the petri dish. Several of the EBV-infected NP outgrowths have by now developed into cell strains after many subcultures over a period of up to 1 year. Morphologically, the change seen under light microscopy was equally remarkable. After the first week, the cells from the uninfected NP explants consisted of predominantly polyhedral cells of relatively uniform morphology, with a low nucleus-to-cytoplasm ratio and predominantly normochromatic nuclei (Fig. 2). In contrast, those from the infected NP explants showed marked cellular pleomorphism, increased mitotic indices, abnormal mitotic figures, occasional presence

TABLE.—*Growth of Test and Control Tissue Explants Observed at the 15th Day after Infection with an EBV Preparation from B95–8 Cell Line*

| Tissue | No. of specimens* | Mean diam. growths (mm ± s.d.) Uninfected | Infected | Mean growth ratio infect./uninfect. ±s.d. | $P$† ($t$ test) | No. with stimulation ≥ 1·8 /Total | $P$† ($\chi^2$ test) |
|---|---|---|---|---|---|---|---|
| NP | 20 | 12·25±2·88 | 34·55±14·75 | 2·83±1·16 | | 19/20 | |
| NPC | 10 | 15·70±3·23 | 20·30±7·83 | 1·33±0·50 | <0·001 | 3/10 | <0·001 |
| OT | 7 | 14·00±4·16 | 23·00±13·84 | 1·65±0·94‡ | <0·02 | 2/7 | <0·005 |
| Tonsil | 9 | 23·78±5·14 | 26·67±7·35 | 1·14±0·33 | <0·001 | 1/9 | <0·0005 |

* Six uninfected and 6 B95–8-infected explants from each specimen were studied (see text).
† All comparisons were with NP explants.
‡ The large s.d. was due to 1 specimen with a growth ratio of 3·64, whilst the range of the values observed with the other specimens in the same group was between 1·0 and 2·0.

FIG. 2.—High power view of stained outgrowth from an uninfected NP explant 2 weeks in culture. Note mosaic pattern, uniformity of cell morphology, low nucleus-to-cytoplasm ratio and normochromatic nuclei.

FIG. 3.—High power view of stained outgrowth from an EBV-infected NP explant. Note cell pleomorphism, high nucleus-to-cytoplasm ratio and hyperchromatic nuclei.

of multinucleate giant cells, increased nucleus-to-cytoplasm ratio and nuclear hyperchromasia in many of the cells (Fig. 3). All these changes were seen throughout the outgrowths.

### DISCUSSION

The rapid and infinite cell proliferation with formation of foci of cell piling, together with changes in cell morphology, observed after the NP explants had been exposed to the EBV preparation, are some of the features shown by transformed cells. Work is now in progress to demonstrate whether other features (*e.g.* chromosome abnormality and malignant behaviour on transplantation to athymic nude mice) are also present.

That the growth stimulation and other changes observed with the EBV-infected NP explants might be mediated not by EBV but by contaminants, bacterial or viral, has to be considered. The addition of penicillin and streptomycin to the culture medium might have taken care of most of the bacterial contamination but not the viruses, particularly adenovirus, which is normally prevalent in the upper respiratory tract. Infection by adenovirus usually leads, however, to cytolysis, not growth stimulation. Furthermore, since all the explants were treated under the same conditions, it would be difficult to explain why the EBV-infected NP explants were particularly susceptible to the mediating effects of such possible contaminants and not the others including the NP explants uninfected by EBV. It would appear, therefore, that there is a *prima facie* case for EBV being the mediating factor. Light microscopy has shown that the possibly transformed cells in the EBV-infected NP growths were epithelial in morphology, but this has to be confirmed by electron microscopy now being performed in collaboration with Guy de-Thé of the International Agency for Research on Cancer (IARC). So far, this has been achieved by the demonstration of keratin fibrils and desmosomes within the cells of one specimen (de-Thé, personal communication). The cells are also being examined for the presence of EBNA and, in collaboration with Harald zur Hausen of Hamburg, also for EBV-DNA.

The interferon was a generous gift from Dr Jan Vilcek.

This work was supported by World Health Foundation (Hong Kong) and The Hong Kong Anti-Cancer Society.

### REFERENCES

Adams, A. (1973) Concentration of Epstein–Barr Virus from Cell Culture Fluids with Polyethylene Glycol. *J. gen. Virol.*, **20**, 391.

Desgranges, G., Wolf, H., De-Thé, G., Shanmugaratnam, K., Cammoun, N., Ellouz, R., Klein, G., Lennert, K., Munoz, N. & zur Hausen, H. (1975) Nasopharyngeal Carcinoma. X. Presence of Epstein–Barr Genomes in Separated Epithelial Cells of Tumors in Patients from Singapore, Tunisia and Kenya. *Int. J. Cancer*, **16**, 7.

Glaser, R., De-Thé, G., Lenoir, G. & Ho, J. H. C. (1976) Superinfection of Epithelial Nasopharyngeal Carcinoma Cells with Epstein–Barr Virus. *Proc. natn. Acad. Sci., USA*, **73**, 960.

Huang, D., Ho, J. H. C., Henle, W. & Henle, G. (1974) Demonstration of Epstein-Barr Virus-associated Nuclear Antigen in Nasopharyngeal Carcinoma Cells from Fresh Biopsies. *Int. J. Cancer*, **14**, 580.

Klein, G. (1973) *The Epstein–Barr Virus. The Herpesviruses.* Ed. A. Kaplan. New York: Academic Press, p. 521.

Klein, G., Giovanella, B., Lindahl, T., Fialkow, P. J., Singh, S. & Stehlin, J. (1974) Direct Evidence for the Presence of Epstein–Barr Virus DNA and Nuclear Antigen in Malignant Epithelial Cells from Patients with Anaplastic Carcinoma of the Nasopharynx. *Proc. natn. Acad. Sci., USA*, **71**, 4737.

Trumper, P. A., Epstein, M. A. & Giovanella, B. C. (1976) Activation *In vitro* by BUdR of a Productive EB Virus Infection in the Epithelial Cells of Nasopharyngeal Carcinoma. *Int. J. Cancer*, **17**, 578.

Wolf, H., zur Hausen, H. & Becker, V. (1973) EB Viral Genomes in Epithelial Nasopharyngeal Carcinoma Cells. *Nature, New Biol.*, **244**, 245.

Wolf, H., zur Hausen, H., Klein, G., Becker, V., Henle, G. & Henle, W. (1975) Attempts to Detect Virus-specific DNA Sequences in Human Tumors. III. Epstein–Barr Viral DNA in Non-Lymphoid Nasopharyngeal Carcinoma Cells. *Med. Microbiol. Immunol.*, **161**, 15.